# Resolving bulimia nervosa using an innovative neural therapy approach: two case reports

**DOI:** 10.1002/ccr3.1326

**Published:** 2017-12-22

**Authors:** Michael I. Gurevich, Myung Kyu Chung, Patrick J. LaRiccia

**Affiliations:** ^1^ Psychiatry and Integrative Medicine 997 Glen Cove Avenue Glen Head New York 11545; ^2^ Cooper Medical School of Rowan University Chung Institute of Integrative Medicine 110 Marter Avenue, Suite 507 Moorestown New Jersey 08057; ^3^ Center for Clinical Epidemiology and Biostatistics Perlman School of Medicine University of Pennsylvania Philadelphia Pennsylvania; ^4^ Penn‐Presbyterian Medical Center Won Sook Chung Foundation 51 N. 39th St. Philadelphia Pennsylvania 19104

**Keywords:** Bulimia nervosa, injections, local anesthesia, neural therapy

## Abstract

Conventional treatment of Bulimia Nervosa is long term, expensive, and often ineffective. Neural therapy holds promise for treating Bulimia Nervosa in a shorter term, lower cost, and more effective manner. Much of neural therapy involves the superficial injection of local anesthetic injections. Implementation into current practice would be feasible.

## Introduction

Bulimia Nervosa (BN) is characterized by binge eating and compensatory behavior such as frequent self‐induced vomiting 1. Lifetime prevalence is 1% worldwide 2, affecting women at 1.5% and men at 0.5% 2. BN is often accompanied by additional psychopathology, suicidal behavior 3, and physical complications 2, 4. Treatment for BN is complex: involving teams of practitioners 5 including group, family 6, and individual psychotherapists; dietary consultations; medical management of complications; psycho‐pharmacotherapy; hospitalizations and day treatment programs 7, 8. However, treatment efficacy remains limited, with high dropout rates, frequent relapses, and long‐lasting morbidity 5, 6, 7, 8.

We present two case reports of patients with BN who had failed conventional medical treatment. Their treatment regime included an innovative component called neural therapy (NT). This approach appears to have provided rapid recovery of cases in my practice (MG) and a possible new outlook on the pathophysiology of the condition.

Neural therapy 9 (NT) is a method that uses injections of local anesthetic into trigger points, scars, peripheral nerves, autonomic ganglia, tendon and ligament insertions, the epidural space, and tissues. The determination of injection site(s) depends on the findings of a conventional medical evaluation in the context of referred pain, dermatomes, regional influence of autonomic ganglia, and identified interference fields (IFs). IFs can be generated by any damaged tissue via chronic stimulation of afferent neurons in the autonomic nervous system resulting in chronic autonomic reflex activity such as nausea, vomiting, and pain. Scars are often sources of interference fields.

NT was developed in Germany and the former USSR in the first half of the 1900s. It is commonly used in German‐ and Spanish‐speaking countries, but poorly known in the English‐speaking world. It has been used for the treatment of multiple medical conditions, injuries, and pain 10, 11, 12, 13, 14, 15, 16, 17, 18, 19, 20, 21. However, its application in the treatment of psychiatric conditions is largely unexplored. The research from VA hospitals to treat veterans suffering from PTSD using Stellate Ganglion injections 22, 23 (which is a NT technique) shows very promising results. It has been used as an adjunct therapy for bipolar disorder patients with good results 24, 25. A literature search in PubMed (which includes MEDLINE), EMBASE, AMED, and CINAHL found no prior report of the use of NT in the treatment of BN. Both patients mentioned herein have provided signed written consent for publication of this report.

## Case Presentations

A 48‐year‐old married female executive presented with persistent daily vomiting for the last 20 years. She self‐induced vomiting nightly. It was a compulsive habit which she accepted to maintain stable weight, reduce guilt from eating large portions at night, and relieve uncomfortable sensations in her stomach. She had suffered from bulimia and anorexia since age 16. At that time, she required extensive therapy managing distorted body images, excessive and compulsive food consumption, and vomiting to control her weight. Over the last several years, her weight had been stable on a highly restricted diet. But any significant bending forward caused her to vomit. She reported an inability to feel emotions and cry. She was sexually abused at age 11 and abused drugs and alcohol in her late teens. Her father committed suicide when she was 6 years old. He suffered from alcoholism and depression. She tried multiple conventional therapies but felt limited or no improvement. Despite her psychiatric issues, she excelled academically and professionally.

Her past medical history included insomnia, depression, hypothyroidism, gastro‐esophageal reflux, and other digestive problems. She received multiple courses of antibiotics due to frequent infections and food poisonings. She ingested two glasses of wine nightly to cope with insomnia. She was taking medications: Escitalopram, Ambien, Levothyroxine, Famotidine, Omeprazole, and Calcium Carbonate. Her initial DSM5 psychiatric diagnoses were as follows: Anorexia Nervosa in full remission, Bulimia Nervosa, moderate; PTSD; substance use disorder in full remission; and major depression, and mild. Her Clinical Global Impression‐Severity (CGI‐S) score was 5.

Neural therapy injections using a total of 10 mL of preservative‐free 1% procaine were injected into the following locations: scar on the right side of the face—1 mL; the umbilical scar—2 mL; intracutaneous blebs over the area of the esophagus—2 mL; xyphoid process—1 mL; upper abdomen—2 mL; and periosteal injections into the sternum—2 mL. During injections, she was asked to focus on her emotional traumas, losses in her life, and her compulsion to vomit resulting in strong emotional relief with a lot of tears.

Also, a modified form of EMDR (eye movement desensitization and reprocessing) was performed to facilitate processing of emerging emotions. She was asked to follow with her eyes MG's hand making figure eights in the air, while focusing on stressful events resulting in further emotional release. That night she did not vomit.

The patient's follow‐up appointment occurred 2 weeks later. She had discontinued Famotidine, Omeprazole, and Calcium Carbonate, and later tapered off Escitalopram. There has not been a single episode of self‐induced vomiting or vomiting associated with bending forward for the last 23 months of follow‐up. Her CGI‐S score is 1. She has had at minimum monthly follow‐up visits for other health concerns and prevention. Currently, she has weekly to bi‐weekly appointments with an acupuncturist/naturopathic physician in MG's office.

### Case #2

A 25‐year‐old white male presented with a chief complaint of 5 months of self‐induced vomiting and 30‐pound weight loss. Six months prior, he had discontinued a gluten‐free diet and began to indulge in rapidly consuming large amounts of junk food, and hiding this behavior from his family. He felt guilty and fearful of gaining weight and becoming fat. He resolved his fear and guilt by self‐induced vomiting after each binging episode. The number of vomiting episodes quickly progressed to several times per day, becoming compulsive and unavoidable.

During vomiting episodes, he experienced emotional relief and almost sexual‐like pleasure akin to a drug addiction. Initially, he felt gratified for controlling his weight during binging episodes but soon lost control, being unable to resist urges to vomit. This compulsion reminded him of the struggle he experienced earlier in his life coping with drug abuse. Compulsive behavior escalated over the preceding 2 months. He was anxious and preoccupied with the need to vomit, which he induced after each meal. He began to lose weight rapidly. His parents, concerned about unexplained weight loss, consulted a gastroenterologist. Gastroscopy revealed esophagitis. He was started on Omeprazole with some relief of heartburn and vomiting, but he developed severe constipation.

The patient had long‐standing issues with anxiety and substance abuse. At age 23, he developed psychosis, depression, and suicidal ideations, diagnosed by a consulting psychiatrist as schizophrenia. Several psychotropic medications were prescribed, but his parents refused medication treatment. MG was consulted, changing the diagnosis to drug‐induced psychosis. He was treated using supplements, energetically based psychotherapy, dietary modifications, meditation, and neural therapy. Keeping with the parents’ desires, psychotropic medications were avoided. The patient had 27 office visits over a period of 28 months. He responded well but stopped follow‐up a year and a half before the current visit. His DSM‐5 diagnoses were Bulimia Nervosa, extreme, multiple substance use disorder in full remission, substance‐induced psychotic disorder, in full remission, and generalized anxiety disorder. His Clinical Global Impression‐Severity (CGI‐S) score was 6.

On this current visit, NT injections were applied to the skin overlying the stomach and esophagus ventrally and dorsally; the periosteum of the sternum; and the thyroid gland. Twelve injections were given using 0.5 to 1 cc of preservative‐free 1% procaine, in addition, 3 cc of preservative‐free 1% procaine was given intravenously as an IV push of 1 to 3 min. The patient was also placed on multivitamins, minerals, and omega 3 oils for general support; St. John's Wort and Avena Sativa for symptoms of depression; Soluna™ homeopathic‐phytotherapy remedies # 3,8,17 and 20 for detoxification; and Biotonic™Adaptogenic Tonic for adrenal support, to improve his digestion, decrease inflammation, and assist with anxiety and depression. On a visit 1 month later, he reported cessation of bulimic episodes after the NT injections. His desire to compulsively vomit stopped. However, he could eat only small doses of food, experienced constipation, and indigestion, but was able to gain 2 lb. He continued Omeprazole for another month.

Neural therapy with localizations similar to the initial visit was repeated four times over the next 2 months in visits spaced 2–3 weeks apart. For the following 9 months, the patient was then seen monthly with the main therapeutic emphasis placed on persistent digestive issues, which resolved after a therapeutic trial of antiparasitic medications, a gluten‐free diet, and use of a mindful eating approach. In a total of 18 months of follow‐up, the patient has been free of self‐induced vomiting for 12 months. He had two episodes of self‐induced vomiting at month six related to stress at work and persistent indigestion. At 9 months of follow‐up, his CGI‐S score was 1 and Clinical Global Impression‐Improvement (CGI‐I) score was 1.

## Discussion

Two adult cases of BN responded very quickly to outpatient therapy which included NT. A 48‐year‐old female responded after one NT treatment session and has been free of BN for the last 23 months. A 29‐year‐old male's self‐induced vomiting was eliminated after the first visit. He had two vomiting episodes at the 6‐month mark and has had no vomiting episodes the past 12 months.

The main limitations of this report are the small sample size, its retrospective nature with the possibility of selection bias, and the lack of controlled replication. Case reports sit on the bottom of evidence‐based medicine's hierarchy of study designs. One cannot generalize from such a small sample. Also, other treatments such as EMDR, food supplements, and gluten restriction were administered, thus there is the problem of confounding which includes the possibility that the whole treatment package rather than NT alone was responsible for the therapeutic benefit. Based on our clinical experience and training, we feel that NT contributed the most to the positive outcomes. More vigorously designed future studies are needed to determine the contributions of the individual components and the entire regime as a whole.

Our total experience with BN includes seven cases. The other five cases were treated with psychotherapy, medications, and food supplements without success. Our positive experiences of NT after addition to our practices across all encountered medical problems prompt us to think that NT was a major contributor to the successful outcome of these two reported cases of BN.

BN treatment approaches encompass the application of behavioral modification, dietary changes, and medication management for patients who are often reluctant to accept any extended therapy. Treatment strategies require long‐term management, efforts of multispecialty treatment teams, expensive hospitalizations, and day treatment program management. High dropout rates are common, and full recovery remains problematic. There are no tactics that can offer speedy symptom resolution, are inexpensive, and do not require long‐term compliance or patient commitment.

NT may offer a fresh understanding of BN's pathophysiological mechanisms and a highly effective treatment. It presents the possibility of a rapid resolution of compulsive vomiting and obsessive food behavior.

The pathophysiological mechanism of BN is complicated. A psychologically vulnerable population distressed by substance abuse, sexual and emotional traumas looks for relief from pain and suffering. Indulging in food offers a short‐lasting pleasure. It is our hypothesis that self‐induced vomiting coupled with food ingestion creates a Pavlovian conditioned response. After a number of repetitions, vomiting can become an independent provider of endorphin release, creating a vicious cycle. Part of this cycle is localized in the autonomic nervous system (ANS) within ganglions controlling the stomach and esophagus. Vulnerable tissue becomes part of an interference field (IF). The IF in the damaged esophagus and stomach has a memory of its own: Vomiting creates a pleasure experience. Thus a vicious cycle, akin to narcotic seeking behavior, is formed and maintained by the IF.

The IF concept was developed to explain some of the of the NT effects. Scars, fractures, and dental lesions can create ANS disturbances that can affect other organs. Signals from them can provide nonlinear and nonanatomically connected interference with affected organs. Over the past 90 years, several reports have documented clinical experience suggesting that injecting an IF such as a scar can resolve pain, organ dysfunction, or other symptoms near or at a distance from the IF 9, 10, 11, 12, 13, 14, 15, 16, 17, 18, 19, 20, 21, 22, 23, 24, 25.

Local anesthetic injections, like procaine, have the capacity to repolarize the nerve cell membrane resulting in restoration of organ function 9. Nerve cell repolarization is posited to be the crucial mechanism 9 in contrast to endorphin release in acupuncture.

The rationale for using NT to treat BN is based on the hypothesis that BN is associated with an IF in autonomic nervous system ganglions affecting the esophagus and stomach. The ectodermal layer of the skin is connected to the ANS ganglions controlling those organs. By injecting the ectodermal layer of the skin, the organ function becomes restored. Thus symptoms of compulsive vomiting are eliminated or significantly reduced. “Lightening reaction” a term developed by NT founders denotes an immediate healing reaction. It was observed in both presented cases. Compulsive vomiting stopped immediately in both cases; however, in the second case, there were two additional episodes of vomiting reported 6 months after the treatment.

NT can be convenient for psychiatric patients: It does not require conscious patient participation, examination of underlying psychodynamic mechanisms, or long‐term medication use. Interactions between physician and patient can be relatively brief, and frequent long‐term follow‐up visits are not required. Materials used in NT are inexpensive. Training to learn superficial injections is relatively brief. The method is remarkably safe with very rare side effects. It can be easily incorporated into any treatment program. In my (MG) psychiatric practice, I have witnessed profound effects in treatment‐resistant patients 24, 25.

## Summary

The effectiveness of conventional BN treatment has been problematic. The two reported cases of successfully treated BN patients point to the possibility of NT being an effective therapeutic agent in the treatment of BN. In both cases, patients were able to rapidly halt vomiting. There were no reported adverse events. Future rigorously designed studies are indicated. We are open to collaboration in this endeavor.

## Authorship

Michael I Gurevich, MD: wrote the first draft as the treating physician. Myung Kyu Chung, MD: critically reviewed all aspects of the manuscript with special attention to the description of the basic mechanism of action of NT in the introduction; the description of the neural therapy techniques used in the case presentations; and the proposed mechanisms of action described in the discussion section as a collaborating colleague, neural therapy practitioner, and lecturer. Patrick J LaRiccia, MD, MSCE: engaged in the development of the discussion section, the literature review directly related to NT, content development of the introduction, and editing and formatting the content of the case presentations.

## Conflict of Interest

None declared.
